# Supplementary figures and images for: Small Molecule Cocktails Promote Fibroblast-to-Leydig-like Cell Conversion for Hypogonadism Therapy
Source: Pharmaceutics. 2023 Oct 13;15(10):2456. doi: 10.3390/pharmaceutics15102456 (PMC10610100; doi:10.3390/pharmaceutics15102456)

# Figure S1

**A**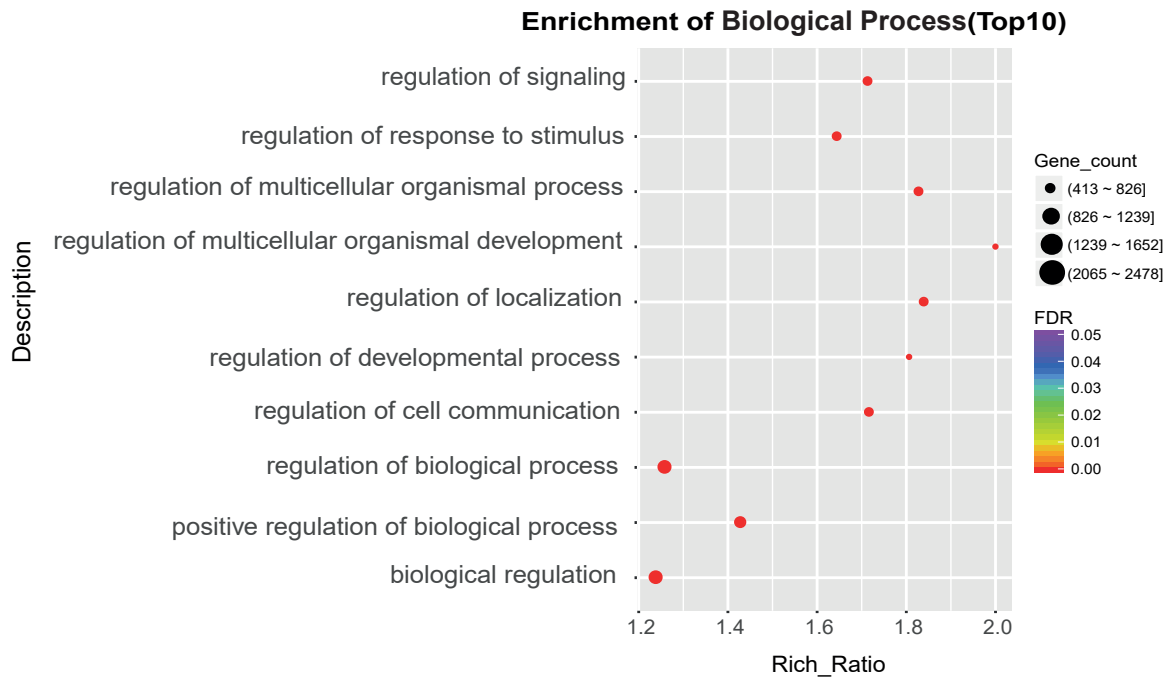**B**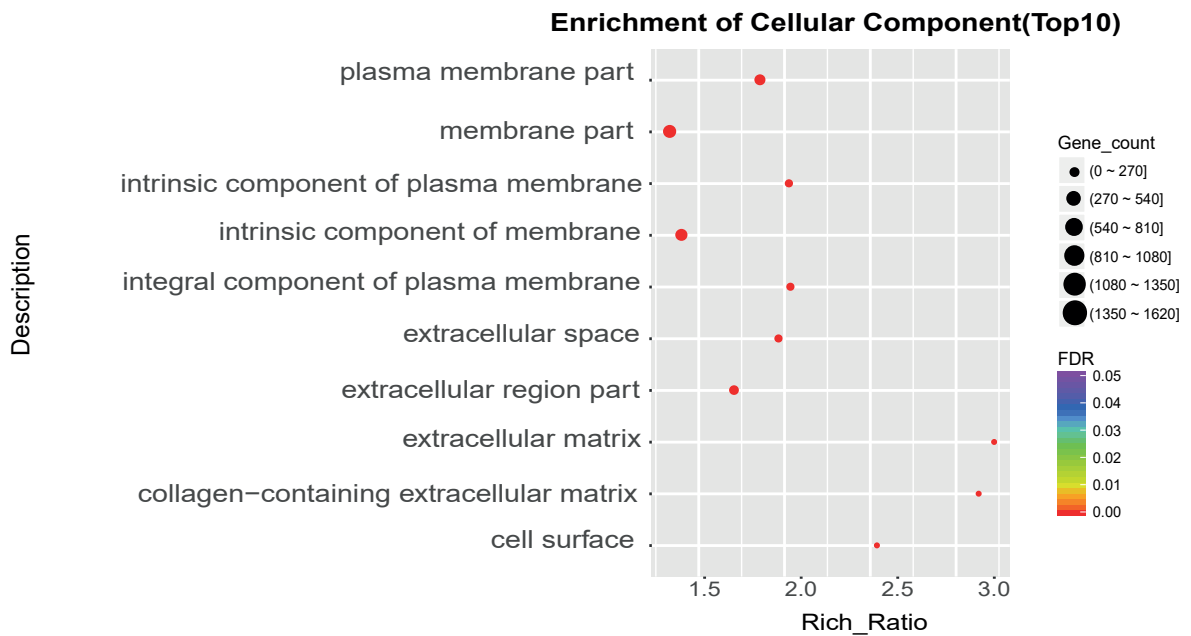**C**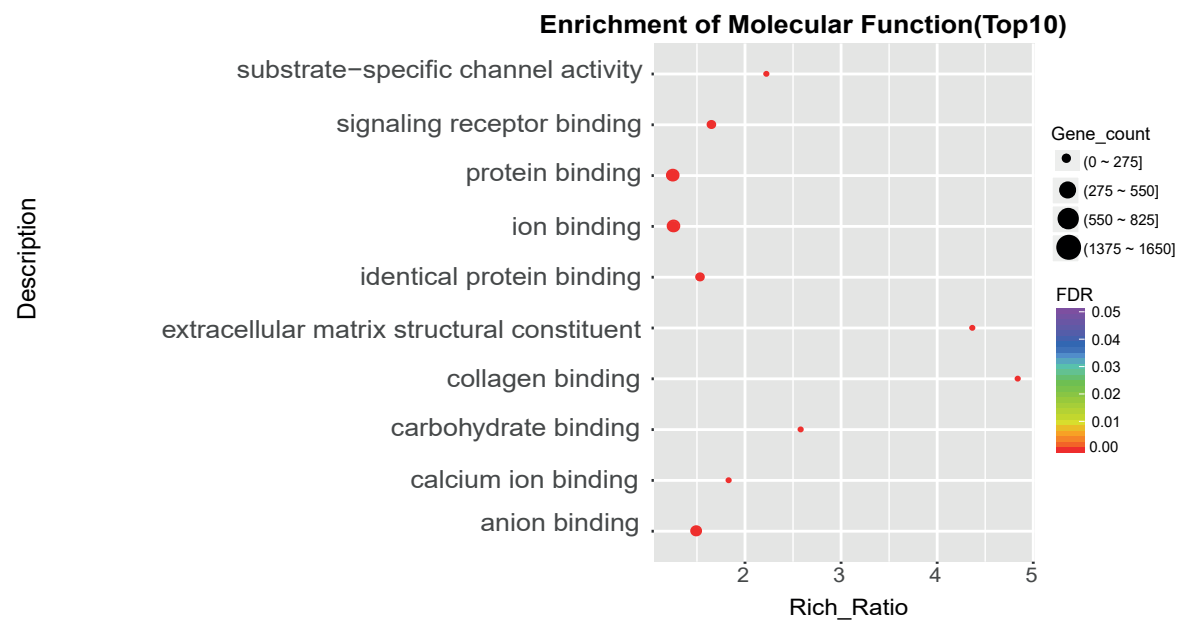

Supplement: Supplementary file 1 [file pharmaceutics-15-02456-s001.zip › Figure S1/Figure S1 .pdf]

Figure S2

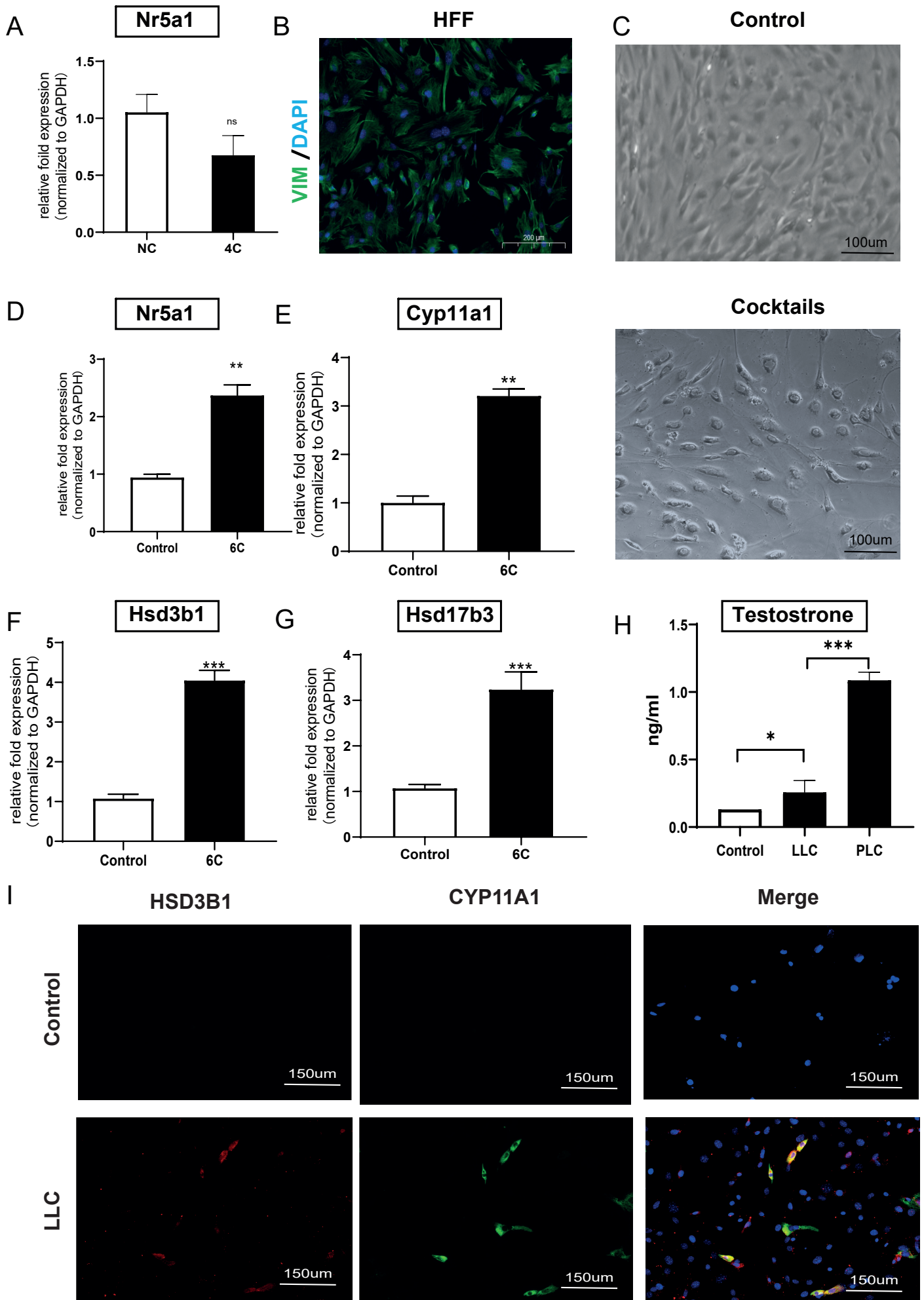

Supplement: Supplementary file 1 [file pharmaceutics-15-02456-s001.zip › Figure S2/Figure S2.pdf]

**Figure S3**

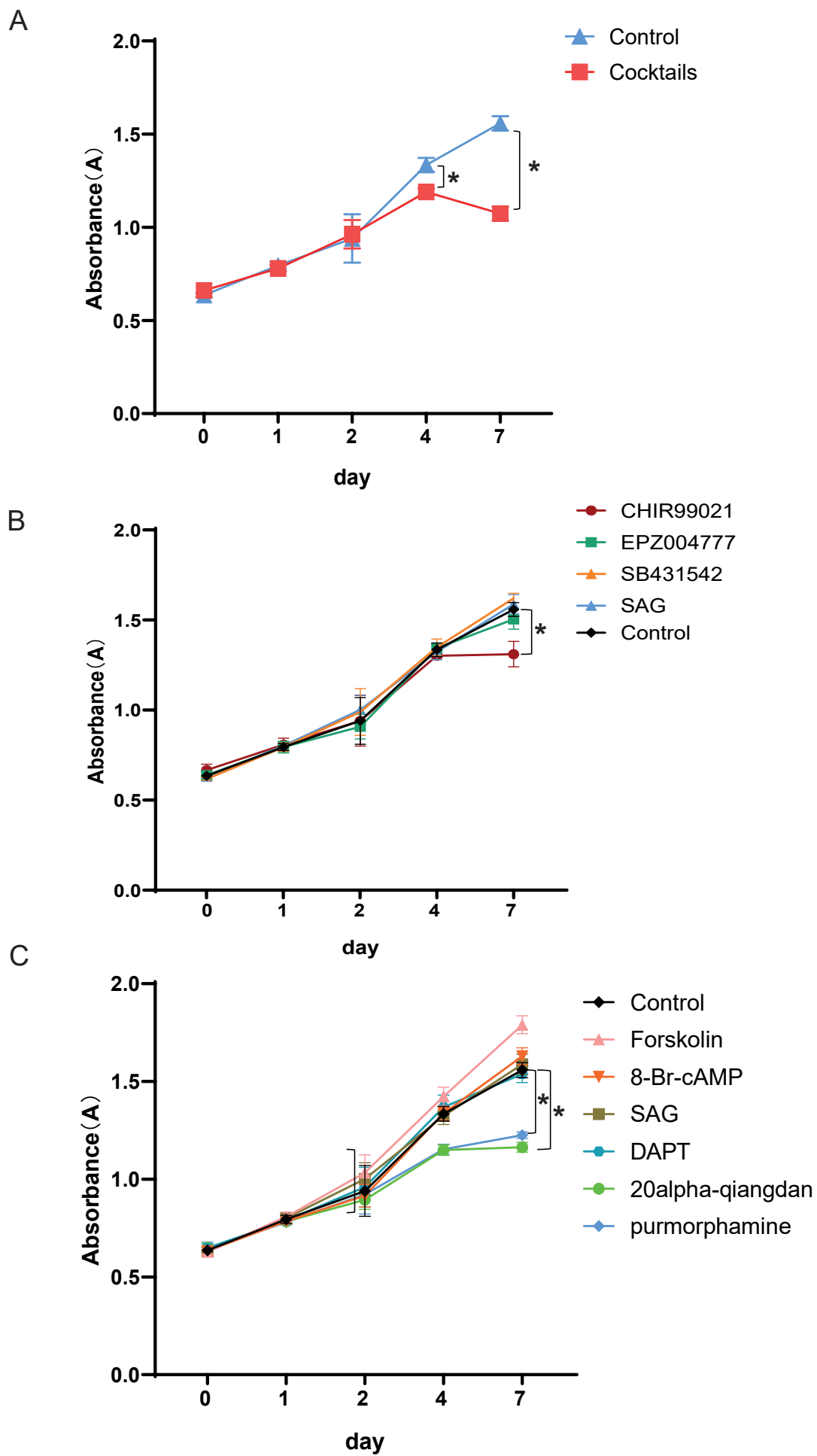

Supplement: Supplementary file 1 [file pharmaceutics-15-02456-s001.zip › Figure S3/Figure S3.pdf]
